# The challenges of open data sharing for qualitative researchers

**DOI:** 10.1177/13591053241237620

**Published:** 2024-03-14

**Authors:** Danielle Lamb, Amy Russell, Nicola Morant, Fiona Stevenson

**Affiliations:** 1 UCL, UK; 2 University of Leeds, UK

**Keywords:** epistemology, methodology, open science, qualitative methods, quantitative methods

## Abstract

‘Open Science’ advocates for open access to scientific research, as well as sharing data, analysis plans and code in order to enable replication of results. However, these requirements typically fail to account for methodological differences between quantitative and qualitative research, and serious ethical problems are raised by the suggestion that full qualitative datasets can or should be published alongside qualitative research papers. Aside from important ethical concerns, the idea of sharing qualitative data in order to enable replication is conceptually at odds with the underpinnings on most qualitative methodologies, which highlight the importance of the unique interpretative function of the researcher. The question of whether secondary analysis of qualitative data is acceptable is key, and in this commentary we argue that there are good conceptual, ethical and economic reasons to consider how funders, researchers and publishers can make better use of existing data.

## Introduction

The ‘Open Science’ movement advocates making scientific research open and accessible to all. As well as encouraging open access to scientific papers, ‘Open Science’ often refers to freely sharing analysis plans, data and analysis code in order that results can be replicated by others, included in meta-analyses, and to identify and avoid fraudulent practices. In quantitative paradigms this is now widely seen as best practice, and there are a number of tools such as the Open Science Framework that support this.

Many funders require project outputs to be published under open access terms (UKRI, 2023), and journals often ask for data and analysis code to be made available in a repository (Kim et al., 2020). However, while wider accessibility of research outputs is relatively uncontroversial, the suggestion that data should be shared for ‘replication’ purposes is at odds with many qualitative paradigms and raises important ethical challenges.

In this commentary we will: set out the background of the open science movement; explore conceptual and ethical concerns; discuss situations in which secondary analysis of qualitative may be appropriate; and suggest ways in which researchers, journals and funders can helpfully respond to the challenges that open qualitative data sharing poses.

We acknowledge that the capitalised term ‘Open Science’ is taken by some to refer to, and give precedence to, quantitative research. As such, for the remainder of this commentary we use ‘open science’ in order to be more inclusive, though we recognise that that some qualitative researchers do not perceive themselves to work within scientific disciplines.

## Background

The open science movement was, at least in part, a response to the ‘replication crisis’ in psychology (Anvari and Lakens, 2018). It refers to the phenomenon whereby initial results about an intervention or psychological construct were significant, while later replications of the same studies found non-significant results. A pivotal replication effort involving attempts to replicate 100 studies found only 36% had significant effects in line with the originals (Open Science Collaboration, 2015). The suggestion is that, ‘In principle, all reported evidence should be reproducible. If someone applies the same analysis to the same data, the same result should occur’ (Nosek et al., 2022). While this may be true of quantitative data, the assumption underpinning this statement of a fixed and single truth is at odds with the epistemologies and associated methodological processes that characterise many forms of qualitative research.

## Conceptual challenges

Replicability has its roots in a realist epistemology. However, while there are many different epistemological stances taken by qualitative researchers, a qualitative approach generally does not seek particular truths or laws of social behaviour, but recognises that knowledge is both situated and created through an interaction of researcher and researched. Qualitative approaches to analysis are typically based on a notion that this involves interpretative work and can therefore generate multiple understandings depending on the theoretical lens through which data is viewed. The suggestion that, for example, a reflexive thematic analysis could be ‘replicated’ is in opposition to these core assumptions, as such an analysis requires the sustained engagement of the unique researcher with the data and its interpretation (Braun and Clarke, 2021).

While other researchers could apply the same analytic techniques to a data set, the resulting analysis would, by definition, not be a replication of the original, as other researchers would bring their unique interests, beliefs and experiences, which would guide their novel interpretations of the data. This was illustrated empirically by Armstrong et al. (1997) who sent the same focus group transcript to a six experts in qualitative data analysis to identify themes. The results showed close agreement on basic themes but each analyst ‘packaged’ the themes differently. This aligns with the recent suggestion that qualitative researchers should provide sufficient contextual and positional information to enable ‘re-renderability’, that is, enable readers to understand how and why the authors have rendered their interpretations, and allow new researchers to ‘re-render’ the data in their own way (Hanchard and Pineda, 2023). We therefore contend that the sharing of full qualitative datasets such as transcripts from interviews for the purposes of *replicating* research findings is not, and should not be, the purpose of open science as applied to qualitative research.

If replication is not (and cannot be) the aim for qualitative research, what does ‘open science’ mean in a qualitative context? As well as addressing the (quantitative) issue of replicability, the benefits of open science are often framed in a narrative of transparency of methods. One sense of transparency is already a key part of many qualitative methods, namely reflexivity. When qualitative researchers reflect on how their own experiences might have influenced an analysis they are being more transparent, arguably, than most quantitative researchers are about their own potential biases in how and why they have analysed data in a particular way. Some qualitative papers include full coding structures and detailed quotes (e.g. as supplemental files), allowing the reader a similar level of insight to the researchers’ workings as the sharing of quantitative statistical analysis code.

The issue that remains is whether there is any reason for journals and funders to require the provision of full qualitative data sets in order to be transparent. It is difficult to see how access to a full data set, for example, of interview transcripts, could increase transparency in a meaningful way (Pownall, 2022). That is, a new researcher given such access would not be checking the accuracy of the original interpretation, given the caveats above about the uniqueness of each individual researcher’s interpretation of data: within a qualitative paradigm, there is no one ‘truth’ to be accurate about.

Conceptually, then, we argue that it is not appropriate or necessary for qualitative researchers to make full data sets publicly available. In addition to these conceptual considerations, there are also ethical concerns with sharing qualitative raw data.

## Ethical challenges

Researchers often do not currently ask for participants’ consent to share entire interview transcripts, and so in those cases making transcripts available beyond the research team of the current project is not ethically permissible. An important reason for only obtaining consent to share excerpts in papers is that participants may be less likely to agree to take part in research if they know entire transcripts will be shared, and may modify what they talk about to researchers if they do take part. However, we do not currently know enough about how making qualitative data sets more open may impact on research participation or data obtained, though work is underway to explore this (Stevenson and Leydon, 2022).

Part of the reason people may be reluctant to consent to full transcripts being made public concerns anonymity. There is a risk of participant identification in qualitative research, even when complete de-identification of transcripts is done (Pascale et al., 2022). It is not always possible to anonymise qualitative data in the way that is possible for quantitative data as each participant’s data consists of a uniquely personal account. This may particularly be the case for narrative research in which individual life stories may be shared (Campbell et al., 2023). Just as quantitative researchers may decline to provide data where very small numbers are involved because it would make participant identification possible, qualitative researchers face the same problem, that is, small numbers of participants, providing data about specific topics or services, may be easily identified. Anonymising qualitative data can be challenging, as context, phrasing, language use or specific personal situations can sometimes easily identify participants, even where names, locations and other obvious identifiers have been removed. There may be options to change or omit potentially identifiable details, but researchers should consider the implications of this for the integrity of data that may be used for secondary analysis.

Related to the importance of anonymity, political and legal contexts can shift dramatically (Keskitalo, 2022; Prosser et al., 2021). This could mean that transcripts about contentious issues, for example, abortion, that are legal at the time of interview could become problematic if, as has happened in the USA, the legal context around that issue changes after publication. There are also shifts in acceptability of language, particularly evident in issues such as race, ethnicity and sex and gender, where attitudes presented in the living past would be perceived as unacceptable now. Changes in confidentiality legislation also raise challenges for researchers deciding what aspects of their data to share (Keskitalo, 2022).

These ethical considerations, together with the conceptual issues outlined above, suggest that the sharing of full qualitative data sets is never appropriate, but is this true?

## Appropriateness of secondary qualitative data analysis

Some have indeed argued that secondary analysis of qualitative data should never be undertaken, citing epistemological and methodological reasons, that is, only those who collected the data will have full understanding of the context (Chauvette et al., 2019; Mauthner et al., 1998). However, while data collectors may well have important contextual knowledge, they do not hold the only important knowledge – theirs does not preclude others from having equally important knowledge. Indeed, the suggestion that only the data collector has the appropriate knowledge and understanding to analyse and interpret the data suggests that their interpretation is the only accurate or acceptable one, an assumption that is at odds with a key tenet of qualitative research regarding plurality of understandings. Aside from the reuse of individual data sets, there is existing good practice around metasynthesis of qualitative data, and many examples of this (Billings et al., 2021; Nicholls et al., 2022; Yarker et al., 2022).

There are also several positive arguments for the reuse of data, and there are many such analyses that have made use of existing qualitative data sets (e.g. Bontempo et al., 2022; Ghio et al., 2021; Ip et al., 2020). From an economic perspective qualitative data is time consuming and expensive to collect, and therefore maximum use should be made of it once it has been collected, particularly where research has been publicly funded. Similarly, from an ethical perspective, participants give their time, share their thoughts and experiences, and often expend emotional labour if topics are sensitive or personally significant, so making best use of the data they provide is the right thing to do (though obviously only if consent is given for others to access their data). Given that qualitative research questions are typically broad with primacy given to how participants themselves frame the topic and decide what is relevant, data sets often include multiple topics or issues that may not have been anticipated by the original research team, but which warrant further analysis. Furthermore, certain populations can be over researched and experience research fatigue, which could be somewhat reduced with the sharing of their data.

Typically, consent is given for a research team to access data, given that it is often the case that multiple researchers will be involved in a study and analysis may be collaborative, so the idea that others may access data is not inherently inappropriate. It is also becoming more common place to include a consent item allowing new researchers to access the data, in consultation with the original research team. More unusually, there are examples of consent items requesting explicit consent for full transcripts to be stored in repositories, with access granted to bona fide researchers via a rigorous checking system. As such, as long as relevant contextual information is provided about the project for which data were collected, the approach to data collection and sampling, strengths and limitations of the data collection process, and the secondary analysis researcher’s own knowledge, experience and assumptions are reflected on appropriately, we argue that there are not *inherent* limitations to the secondary analysis of qualitative data. Indeed, as noted above, there are already examples of best practice in terms of qualitative data repositories such as those provided by the UK Data Service (2023), and the Healthtalk (2023) collection of video interviews.

## Next steps

We have argued above that rigid requirements for qualitative researchers to make full data sets publicly available are inappropriate, but that there are some convincing reasons to consider whether qualitative data could be made available for secondary analysis (given appropriate consent and vetting procedures).

We suggest that researchers, journals and funders can improve the ways in which qualitative data is accessed and used. Firstly, researchers should consider data sharing when designing consent forms. In particular, when it might be appropriate/possible to include consent to share data for potential future secondary analysis. Consideration should be given to the form that data could be made available to others, and whether data could be added to closed repositories, accessed only by authorised researchers. However, there is a lack of agreement about informed consent for secondary analysis of data, and there is a need for wider engagement about this, in particular involving patients and members of the public.

Secondly, journals that accept qualitative paper submissions should revise data access requirement statements to recognise the methodological differences between quantitative and qualitative research, with no rigid requirements to share full data sets.

Thirdly, funders could support qualitative researchers to make best use of data, without requiring publicly accessible full data sets. This could include funding new research teams to access to existing data if useful research questions can be answered through secondary analysis, and providing funds for the additional time and cost of preparing data to be added to repositories.

In closing, we would point readers to ongoing work in this space, such as the ‘Qualitative Data Preservation and Sharing’ (Q-DaPS) NIHR-funded project that is developing a qualitative data repository (Stevenson and Leydon, 2022), and discussion in forums such as the NIHR Methodology Incubator qualitative workstream (NIHR, 2020) and the UCL Qualitative Health Research Network (UCL, 2018).
